# A systematic review of physical activity policy recommendations and interventions for people with mental health problems in Sub-Saharan African countries

**DOI:** 10.11604/pamj.2017.26.104.10051

**Published:** 2017-02-28

**Authors:** Davy Vancampfort, Brendon Stubbs, Marc De Hert, Christy du Plessis, Caleb Ademola Omuwa Gbiri, Jepkemoi Kibet, Nancy Wanyonyi, James Mugisha

**Affiliations:** 1 KU Leuven – University of Leuven, Department of Rehabilitation Sciences, Leuven, Belgium; 2 KU Leuven - University of Leuven, University Psychiatric Center KU Leuven, Leuven-Kortenberg, Belgium; 3 Physiotherapy Department, South London and Maudsley NHS Foundation Trust, London, UK; 4 Health Service and Population Research Department, Institute of Psychiatry, Psychology and Neuroscience, King’s College London, De Crespigny Park, London, UK; 5 University of the Free State, Bloemfontein, South Africa; 6 College of Medicine, University of Lagos, Nigeria; 7 Department of Psychiatry, Faculty of Health Sciences, University of Pretoria, South Africa; 8 Center for Victims of Torture, Nairobi, Kenya; 9 Department of Orthopaedics and Rehabilitation, Moi University College of Health Sciences, Eldoret, Kenya; 10 Kyambogo University, Kampala, Uganda; 11 Butabika National Referral and Mental Health Hospital, Kampala, Uganda

**Keywords:** Physical activity, exercise, mental health services, Sub-Sahara Africa

## Abstract

**Introduction:**

There is a need for interventions to address the escalating mental health burden in Sub-Saharan Africa (SSA). Implementation of physical activity (PA) within the rehabilitation of people with mental health problems (PMHP) could reduce the burden and facilitate recovery. The objective of the current review was to explore (1) the role of PA within mental health policies of SSA countries, and (2) the current research evidence for PA to improve mental health in SSA.

**Methods:**

We screened the Mental Health Atlas and MiNDbank for mental health policies in SSA countries and searched PubMed for relevant studies on PA in PMHP in SSA.

**Results:**

Sixty-nine percent (=33/48) of SSA countries have a dedicated mental health policy. Two of 22 screened mental health policies included broad physical activity recommendations. There is clear evidence for the role of PA in the prevention and rehabilitation of depression in SSA.

**Conclusion:**

Despite the existing evidence, PA is largely a neglected rehabilitation modality in the mental health care systems of SSA. Continued education of existing staff, training of specialized professionals and integration of PA for mental health in public health awareness programs are needed to initiate and improve PA programs within the mental health care systems of SSA.

## Introduction

Mental and substance use disorders are the leading cause of years lived with disability (YLD) in Sub-Saharan Africa (SSA), accounting for 19% of all disability-associated burden (YLD) [1]. Major depressive disorders are the second leading cause of disability following chronic back pain while anxiety disorders the fifth leading cause of disability in SSA. In terms of mental and substance use disorders, all SSA regions will experience an increase in burden of approximately 130% and it is estimated that the YLDs will rise from between 20 to 45 million YLDs by 2050 [2]. Moreover, by 2050 mental and substance use disorders may be equivalent to approximately two thirds the YLDs of the entire non-communicable diseases group (67 million YLDs) of SSA [3]. The consequences of the rising and devastating burden of mental and substance use disorders are far-reaching and long-lasting, not only for the individual but also for the family and community as a whole. The quality of life of those affected is severely impacted and economic costs are significant. It is estimated that the cumulative global impact of mental and substance disorders may amount up to US$16 trillion due to lost economic output over the next 20 years [4]. Moreover, secondary co-morbidities need to be considered which can add to the increased disability and burden [5, 6]. For example, severe mental illness has been shown to be an independent risk factor for other important non-communicable disorders such as cardio-metabolic diseases, albeit inconsistently in SSA studies [7]. Consistent associations are however reported between HIV/AIDS and poor mental health [8, 9] and chronic pain and poor mental health [10, 11]. Also the strong and often bidirectional relationships of mental disorders with substance use disorders and the associated increased risk for accidents and injuries emphasizes the critical role of a rigorous mental health policy in SSA [3].

Given the increasing pressures of communicable diseases and malnutrition, a mental health policy has been relatively low on the priority list in most of SSA countries [12, 13]. Only a median of 0.62% of the health budget is spent on mental health [14]. As a result, mental health services are poorly resourced and typically very few inpatient facilities are available in the larger urban cities [13]. Treatment rates for people with mental disorders remain low, with treatment gaps over 90% [15]. The World Health Organization Comprehensive Mental Health Action Plan for 2013 to 2020 [3] outlines targets for its member countries, which include updating existing mental health policies and plans, integrating mental health care into community-based settings, and strengthening evidence-based research. One major concern of the plan is the limited research in mental health care in the Sub-Saharan region [16]. To date, within low-to-middle income countries, community-based rehabilitation, psychoeducation and support for families (delivered by non-specialists) are recommended for low resource settings, with assertive community care and cognitive therapy recommended as additions in higher resourced settings with stronger service-delivery platforms [17]. The potential role of low-cost physical activity interventions seems to be given low priority and neglected. This is not surprising since the emphasis in health service delivery in SSA is based on the biomedical model (versus the biopsychosocial model) with an important focus on pharmacology in the management of mental disorders [13]. As a result, more doctors and nurses are recruited as key cadres as compared to any other cadre of staff and this stifles the promotion of other biopsychosocial packages (including physical activity) in the management of most conditions [13]. There is however an increasing body of research demonstrating that physical activity interventions prevent the onset of mental illnesses such as depression [18] while it can improve physical, mental and social health outcomes in people with established mental disorders [19–22]. Physical activity has also been shown to reduce cognitive deficits [19, 23, 24], aspects of the illness which are often left untreated and particularly influential on long-term functioning [25, 26]. Thus, implementation of physical activity interventions within the care of people with mental health problems could reduce the mental, physical and social burden, while facilitating functional recovery and consequently reducing disability. This will on its turn reduce the societal costs. The aim of the current systematic review is twofold. First, we set out to explore the role of physical activity within the current mental health policies and plans of SSA. Specifically, we wanted to explore whether any priorities or recommendations were reported and in particular explore the integration of health care professionals with potential expertise in delivering physical activity interventions (i.e., physical therapists, physical educators, exercise physiologists and occupational therapists). Second, we explored the current research evidence from physical activity studies performed in Sub-Saharan Africa.

## Methods

### Screening for physical activity priorities and recommendations in mental health policies and plans in Sub-Saharan Africa

In a first stage, we screened the latest Mental Health Atlas [27]. If the country data were not available in the latest edition, the penultimate edition was screened. With data from 171 World Health Organization (WHO) Member States, the Mental Health Atlas provides a comprehensive overview of mental health policies, plans and services worldwide. Data abstracted were: (a) presence of a mental health policy and/or plan (yes or no), and (b) the number of therapists with potential expertise in exercise prescription registered (per 100,000 population). The Mental Health Atlas only reports data on occupational therapists. In a second stage, if a mental health policy and/or plan is available the full-text documents were retrieved via the MiNDbank of the World Health Organization [28]. Policies written in English, French, Spanish or Portuguese were evaluated. If the mental health policy and/or plan was not available google scholar was screened using the search terms: “mental health” AND “plan” OR “policy” and the name of the country, or its equivalents in other languages. Mental health policies and plans were screened for physical activity priorities and recommendations. Search terms used, were: “physical activity” OR “exercise” OR “sports” OR “activities” OR “rehabilitation” OR “active lifestyle” OR “physical health”, or its equivalents in other languages. In a third stage, we summarized the reported priorities and evaluated the quality of the physical activity recommendations, if available. We verified the efficacy and effectiveness of the recommendations. More in detail we explored: (a) whether physical activity priorities and recommendations were based on available scientific evidence (reference made to the scientific literature or not), (b) whether physical activity recommendations were defined in terms of frequency, intensity, the time (in minutes), and the type of physical activity (FITT-principle), and (c) how physical activity interventions should be implemented in daily care (by whom and which motivational approaches are recommended to increase adherence and reduce dropout).

### Identification of physical activity studies conducted in people with mental health problems in SSA

**Search strategy:** We systematically search PubMed from inception until May 1st, 2016 for relevant studies on physical activity in people with severe mental illness in SSA. The following search strategy was used: “physical activity” OR “exercise” OR “sports” OR “rehabilitation” AND “mental” OR “depression” OR “psychosis” OR “schizophrenia” OR “bipolar” AND the name of the country.

#### Eligibility criteria

**Participants:** Although we were interested in people with mental disorders, we did not exclude any people due to age or whether or not they were diagnosed with Statistical Manual (DSM) [29, 30] or International Classification of Disease (ICD) [31] criteria or with other validated diagnostic tools.

**Interventions:** Any physical activity interventions were included. Physical activity was defined as any bodily movement produced by skeletal muscles that results in energy expenditure. Physical activity in daily life can be categorized into occupational, sports, conditioning, household, or other activities. Exercise studies were also included which is a subset of physical activity that is planned, structured, and repetitive and has as a final or an intermediate objective the improvement or maintenance of physical fitness [32].

**Control conditions:** The presence of control conditions was not an inclusion criterion. However, if studies were clinical or randomized controlled, usual-care or wait-list control conditions were included.

**Outcome measures:** The primary outcome measure was any mental health outcome measure. Secondary outcomes were physical health outcomes in mentally ill populations. If available, physical activity and / or sedentary outcome measures were also included.

**Study design:** We included cross-sectional studies and pre- and post-test studies without a control group and randomized (RCTs) or non-randomized clinical controlled trials (NRCCTs) in which the experimental and control intervention were of similar duration.

**Exclusion criteria:** In case of overlap only the most recent study or the study with the largest dataset were included. No additional exclusion criteria were applied.

**Study selection:** Two reviewers (DV and BS) screened titles and abstracts of all potentially eligible articles. Both authors applied eligibility criteria, and a list of relevant studies was developed through consensus. When necessary, the protocol stated that the corresponding author of a study would be contacted up to two times in a 4-week period to request data that would enable inclusion in the current analyses.

#### Data extraction

Two authors (DV, BS) extracted data using a predetermined data extraction form. The data extracted for were country, study setting, patient characteristics (diagnosis, age, % male). Duration (weeks), frequency (times per week), intensity (as defined by the authors), and type (aerobic exercise, resistance training, mixed) of the physical activity intervention, whether the exercise was supervised or not and qualified versus non-qualified providers. Providers of physical activity interventions were considered experts when they had at a minimum a bachelor-level degree in physical therapy, exercise physiology or a similar that included education in exercise prescription and assessment. Finally we did extract physical activity and sedentary assessments and motivational strategies used to improve adherence and reduce dropout.

## Results

### Physical activity priorities or recommendations in mental health policies and plans in SSA

In terms of policy, 69% (=33/48) of SSA countries report having a dedicated mental health policy or plan. Ten policies were not found while one (Sudan) was written in Arabic, and therefore not meeting our inclusion criteria and was not screened. Two (Namibia and Uganda) of 22 screened mental health policies or plans included physical activity priorities or recommendations. None of these recommendations were based on scientific evidence, defined physical activity according to frequency, intensity, type or time, nor defined any implementation strategies. Per 100,000 inhabitants the number of occupational therapists available ranged from 0 (n=20) to 5.36 (Seychelles) An overview of the presence of a mental health policy or plan, the number of occupational therapists involved for each country and the availability of physical activity priorities or recommendations is presented in Table 1, while the physical activity priorities or recommendations are summarized in Table 2.

**Table 1:** Overview of the presence of a mental health policy/plan, the number of occupational therapists, physical activity priorities and physical activity studies in Sub-Saharan African countries (n=48)

Country	Official mental health policy or plan	Occupational therapists in the mental health sector°	Physical activity priorities reported or recommendations available	PubMed search results (potential relevant / obtained)
Angola (2011)	Yes	0.01	No	0/9
Benin (2014)	No	0	/	0/53
Botswana (2014)	Yes	0.64	No	0/35
Burkina Faso (2014)	Yes	0	No	0/20
Burundi (2014)	Yes	0.02	No	0/34
Cameroon (2011)	No	UN	/	0/6
Cape Verde (2011)	Yes	0	No	0/4
Central African Republic (2014)	Yes	0.02	No	0/36
Chad (2011)	Yes	0	NA	0/5
Comoros (2011)	Yes	0	NA	0/62
Congo (2014)	No	UN	/	0/19
Côte d'Ivoire (2014)	Yes	0	No	0/21
Democratic Rep. of the Congo (2011)	Yes	0.001	No	0/5
Djibouti (2014)	No	UN	/	0/1
Equatorial Guinea (2014)	No	UN	/	0/18
Eritrea (2011)	No	0	/	0/222
Ethiopia (2014)	Yes	0	No	0/1
Gabon (2011)	No	0	/	0/16
Gambia (2014)	Yes	0	No	0/16
Ghana (2014)	Yes	0	No	1/99
Guinea (2014)	Yes	0	NA	0/3132
Guinea-Bissau (2011)	No	0	/	0/2
Kenya (2011)	Yes	UN	No	0/174
Lesotho (2014)	No	0.14	/	0/4
Liberia (2014)	Yes	0.05	No	0/11
Madagascar (2014)	Yes	0	No	0/9
Malawi (2014)	Yes	0.01	No	0/48
Mauritania (2011)	Yes	0.06	NA	0/6
Mauritius (2014)	No	UN	/	0/16
Mozambique (2014)	Yes	0.1	No	0/13
Namibia (2014)	Yes	0.55	Yes	0/14
Niger (2011)	Yes	0	NA	0/79
Nigeria (2014)	Yes	0.01	No	3/776
Rwanda (2014)	Yes	UN	No	1/50
São Tomé and Príncipe(2014)	Yes	0	NA	0
Senegal (2014)	No	0.01	/	0/48
Seychelles (2014)	No	5.36	/	0/8
Sierra Leone (2014)	Yes	0	No	0/13
Somalia (2014)	No	UN	/	0/46
South-Africa (2014)	Yes	UN	No	1/1391
South-Sudan (2014)	No	0	/	0/5
Sudan (2011)	Yes	0	Not checked*	0/79
Swaziland (2014)	No	0	/	0/12
Togo (2014)	Yes	0	NA	0/48
Uganda (2014)	Yes	UN	Yes	2/227
United Republic of Tanzania(2011)	Yes	0.009	NA	0/109
Zambia (2014)	Yes	0.04	No	0/39
Zimbabwe (2014)	Yes	0.12	NA	0/91
**Summary**	**69% (33/48) has an official mental health policy**	**Range=0-5.36**	**9% (2/22) have physical activity recommendations incorporated**	**8/7133**

UN=unknown, NA=not available

° per 100,000 population

* plan written in Arabic.

**Table 2:** Physical activity priorities and recommendations in mental health plans of Sub-Saharan African countries

Country	Physical activity priorities or recommendations	Based on scientific evidence	FITT defined	Implementation strategies
Namibia	Promote a healthy lifestyle with regular exercise.	No	No	No
Uganda	Conduct health education and promote activities.	No	No	No

FITT= frequency, intensity, time and type.

### Screening for physical activity studies conducted in people with mental health problems in SSA

#### Search results

Out of 7,133 search hits, 8 potentially eligible studies were retrieved. After applying the eligibility criteria 7 studies [33–39] were included. One study was excluded as it overlapped a study already included in the review [35]. An overview of the search results for each country is presented in Table 3.

**Table 3:** Mental and/or physical health outcomes in physical activity studies in Sub-Saharan Africa

First author	Country	Design	Participants	Physical activity intervention or assessment	Mental and/or physical health outcomes^+^
Aweto 2016 [33]	Nigeria	RCT	18 (32.1±5.4 years) outpatients with HIV; BMI=26.1±1.4 vs 15 controls with HIV with care as usual (30.7±5.8 years); 10♂/33	6 weeks, 3*week, 30min moderate intensity aerobic exercise on a cycle ergometer provided by a physiotherapist	The Beck Depression Index score only reduced significantly in the exercise group [10.3±6.5 vs.3.5±1.3;P<0.001]
Balchin 2016 [34]	South-Africa	RCT	30♂ moderately depressed; mean age=25.4 years, mean BMI=26.9	6 weeks, 3xweek, 60min high vs moderate vs low intensity aerobic exercise; providers unknown	The HAM-D (15.9±1.8 vs. 5.7±5.8 and 16.4±1.4 vs. 6.6±5.0 vs. 17.1±1.2 vs. 11.8±3.9, respectively) and MADRS 12.7±4.0 vs. 7.0±6.7 and 14.4±4.3 vs. 9.0±6.7 vs. 18.8±6.4 vs. 15.0±5.2, respectively) only reduced significantly in the high and moderate intensity aerobic exercise
Asare 2015 [35]	Ghana	Cross-sectional	296 adolescents (boys=150, girls=146); 13-18 years	Physical Activity Questionnaire for Adolescents The Adolescent Sedentary Activity Questionnaire	Significant negative correlation with physical activity independent of sedentary behaviour [CDI (r=-0.78, p<0.001); BIS physical self-worth (r=-0.71, p < 0.001); BIS body dissatisfaction (r=-0.76, p < 0.001)]. Sedentary behaviour significantly associated with CDI (r=0.68, p<0.001). Affluence was a significant contributing factor of sedentary behaviour [t(294)=-7.30, p<0.001]
Fatiregun 2014 [36]	Nigeria	Cross-sectional	1,713 adolescents (boys=766, girls=947); 10-19 years	Self-report participation in sporting activities	Respondents who did not participate in any sporting activities had a higher proportion of depressive symptoms (27.3%) when compared with those who did participate in sporting activities (19.4%; P=0.001)
Richards 2014 [37]	Uganda	RCT	1,462 adolescents in the study (intervention: boys=74, girls=81; wait-list: boys=72;comparison: boys=472, girls= 63); 11-14 years	One 90min training and one 40min football game every weekdelivered by 6 paid staff who selected and trained 32 volunteer adults from the local community to become football and peace-building coaches	Negative effect on DLS when comparing boys intervention vs wait-listed (ES = 0.67 [0.33 to 1.00]) and intervention vs non-registered (ES = 0.25 [0.00 to 0.49]). Idem for ALS for boys intervention vs wait-listed (ES = 0.63 [0.30 to 0.96]) and intervention vs non-registered (ES = 0.26 [0.01 to 0.50]). There was no significant effect on the girls for any outcomes

+ Physical health outcomes only reported in mental health populations; RCT= randomized controlled trial, HAM-D= Hamilton depression score, MADRS=Montgomery-Åsberg Depression Rating Scale, DLS=Acholi Psychosocial Assessment Instrument for local depression-like syndromes, ALS= Acholi Psychosocial Assessment Instrument for local anxiety-like syndromes; CDI= Children’s Depression Inventory; BIS=Body Image Silhouette test.

#### Participants and study characteristics

Four RCTs and 3 cross-sectional studies involving in total 4,737 participants (45.4% male) were included. One RCT (Uganda) and the 3 cross-sectional studies (Ghana and Nigeria=2) included only adolescents (n=4,571). Two RCTs focused on mental health outcomes in people with HIV (Rwanda and Nigeria; n=133), one among depressed adults (South-Africa; n=30) and one on depression and anxiety like syndrome in school children in a post-conflict setting (Uganda; n=1,462). The interventions ranged from 6 to 26 weeks, from 30 to 90 min per week, from 2 to 3 times per week and from low to high intensity. Three RCTs explored aerobic exercise, one a sports-for-development intervention including football. Details of the participants and study characteristics are presented in Table 3. Providers, when reported, were physiotherapists (n=1) or trained community coaches (n=1). In cross-sectional studies, the physical activity behavior was assessed with self-report instruments.

#### Physical activity outcomes

In the 2 RCTs in people with HIV, aerobic exercise reduced depression and improved psychological quality of life, self-esteem, body image and emotional stress. In one RCT in adolescents sports delivered by trained community coaches had a negative effect on depression and anxiety like symptoms in boys while no effect on girls. In one RCT in depressed adults only moderate and high but not light intensity aerobic exercise resulted in significantly less depressive symptoms. When looking at the 3 cross-sectional studies in adolescents all 3 studies consistently showed that less physical activity participation is associated with more severe depressive symptoms. Details of the physical activity intervention characteristics, outcomes and assessment tools are presented in Table 4.

**Table 4:** Mental and/or physical health outcomes in physical activity studies in Sub-Saharan Africa

First author	Country	Design	Participants	Physical activity intervention or assessment	Mental and/or physical health outcomes+
Adeniyi 2011 [38]	Nigeria	Cross-sectional	1,100 adolescents (boys=538, girls=562); 12-17 years	Physical Activity Questionnaire for Adolescents	Higher CDI scores were linked with lower levels of physical activity (r=-0.82, P< 0.001) and moderate physical activity was linked with reduced risk of depressive symptoms (OR = 0.42, 95%CI=0.29-0.71)
Mutimuura 2008 [39]	Rwanda	RCT	50 (20♂) (37.5±6.9 years) outpatients with HIV; 88% employed; BMI=24.4±2.7; 20% smoking vs 50 (20♂) controls with HIV with care as usual (37.8±5.5 years)	26 weeks, 3xweek, 90min moderate intensity aerobic and resistance training; providers unknown	At 6 months, scores on psychological quality of life [1.3±0.3 vs. 0.5±0.1; P<0.0001], self-esteem [1.3 ±0.8 vs. 0.1±0.6); P< 0.001], body image [1.5±0.6 vs. 0.0±0.5; P <0.001] and emotional stress [1.6±0.7 vs. 0.2±0.5; P < 0.001], improved more in the exercise group

+ Physical health outcomes only reported in mental health populations; RCT= randomized controlled trial, HAM-D= Hamilton depression score, MADRS=Montgomery-Åsberg Depression Rating Scale, DLS=Acholi Psychosocial Assessment Instrument for local depression-like syndromes, ALS= Acholi Psychosocial Assessment Instrument for local anxiety-like syndromes; CDI= Children’s Depression Inventory; BIS=Body Image Silhouette test.

## Discussion

The current systematic review shows that 69% of the SSA countries have a mental health policy or plan. When screening the available mental health policies or plans we found that less than 1 in 10 makes reference to the importance of considering an active lifestyle and/or structured exercise. Therefore, although physical activity is becoming acknowledged as a major component in the management of severe mental illness [40, 41], the potential is yet to be embraced in SSA. The lack of priority given to an active lifestyle and/or structured exercise as a complementary treatment is also mirrored in the limited number of studies exploring the importance of physical activity and exercise in the prevention or management of mental health problems. It is plausible that due to the strong focus on pharmacotherapy [13] health care professionals in SSA are yet to be aware of the beneficial effects of physical activity as one of the components of the multidisciplinary treatment of people with mental illness. Hence, the need to re-orient the current health care systems including policy makers to embrace other biopsychosocial approaches in the management of mental disorders is needed. Key staff to promote and deliver such physical activity interventions should at a first stage probably be recruited within the current health systems in SSA. Our review shows that there is until today a lack of adequate providers. In 20 of 37 countries providing data no occupational therapists were involved in the multidisciplinary treatment. Secondly, there is a need to reduce the patient/clinician ratio, which is nowadays a challenge for key staff members as they have to concentrate on only critical curative packages at the expense of some packages such as physical activity. Thirdly, public health training institutions should focus on the importance of physical activity for mental health in order to improve the competencies of their graduates in this field. To this end, the current available studies provide several points of interest which are also in agreement with rigorous meta-analytic findings [19, 21, 42, 43]. For example, the current review shows that aerobic exercise might reduce depressive symptoms in people with mental health problems in SSA. Secondly, our data also show that exercise is an important adjunct in the treatment of people living with HIV confronted with mental health problems. Thirdly, our review consistently shows that in young people feelings of depression are related to a sedentary lifestyle and lack of physical activity. Although the studies in adolescents were executed in non-clinical samples, these findings indicate that early intervention strategies are highly needed, also in SSA countries. More research is needed for the most optimal interventions. The study in Ugandan adolescents in a post-conflict setting [35] clearly showed that sports-for-development programs might result in negative findings. Although further exploration of these findings is warranted it might be assumed that the lack of adequate therapeutic training of the local football and peace-building coaches might be a reason. These findings point again towards the need of adequate training of care providers.

Several strategies to initiate and improve physical activity and exercise programs within the mental health care systems of SSA are possible. For example, continued medical education (CMEs which is a common practice) should be used to re-tool staff on the importance of physical activity. The existing working force should be informed regarding the importance of an active lifestyle for their patients and should be trained in skills to deliver easy implementable interventions. Asking about and advising regarding physical activity might help to improve physical activity uptake or maintenance [44]. One approach that should be tested in SSA is the Physical Activity Vital Sign (PAVS) method. This method may inform patients about the health recommendations but might also enable existing working force to identify those people with mental health problems who are also at risk of metabolic abnormalities. The PAVS is a two-question measure to assess the adherence to the international recommendation of 150 minutes per week of moderate to vigorous physical activity [45]. Recent studies [46, 47] in mental health populations in Belgium clearly demonstrated that those who did not adhere to the minimum physical activity recommendations, as formulated by the PAVS-method, have higher metabolic risks. The brevity of the PAVS, along with the implementation of multidisciplinary care, may help promote the importance of physical activity and exercise assessment and prescription as a core part of the treatment of people with mental health problems in low-resources settings. Each member of the multidisciplinary team can play an important role in encouraging people with mental health problems to engage in routine physical activity. For example, in addition to assessing the usual vital signs, nurses could utilize the PAVS-questions while recording the answers in an individual’s baseline assessment [48]. During the subsequent consultation, a psychiatrist could provide positive reinforcement to patients achieving 150 min of physical activity while advising them to maintain their physical activity behaviour. Those patients who are not managing to achieve 150 minutes per week should be advised to become more active and informed the importance of complying with the guidelines. When time does not permit, or when patients are confronted with severe symptoms and are consequently struggling to be more physically active and/or those who are suffering from cardiovascular, respiratory, neurological or musculoskeletal conditions, may also benefit from further evaluation by a physical therapist or exercise physiologist [49]. The job of raising awareness, ensuring adherence and compliance and adapting exercise to meet the social, environmental, physical and mental constraints should be referred to and handled by these professionals. Policy makers should therefore invest in qualified professionals to optimize mental and physical health improvements according to individual goals and expectations. It is known that inclusion of these professionals reduces dropout [50, 51] and consequently improves outcomes and ultimately will be cost-effective.

To this end, physical therapists or exercise physiologists in SSA countries should be able to follow training in improving their mental health skills. Providers should be able to recognize the signs and symptoms of psychiatric disorders and demonstrate basic knowledge of the causes. The findings in post-conflict Uganda indicate that providers should be trained in basic therapeutic interactions (e.g. communication, attitude) and in basic motivational skills (e.g., motivational interviewing). Research should explore the most optimal form of delivery. For example, effectiveness trials in different cultural settings could explore whether assisting people with mental health problems in fulfilling three universal, psychological needs: (a) the need for autonomy (i.e., experiencing a sense of psychological freedom when engaging in exercise), (b) the need for competence (i.e., ability to attain desired outcomes following the exercise program), and (c) the need for relatedness (i.e., being socially connected when being physically active) will increase the likelihood that they adopt or maintain an active lifestyle [52]. Ministries of health and education in SSA play a critical role in governance and policy development [13]. Physical activity needs to be mainstreamed in existing health policies and strategies at all levels of intervention including primary health care. It is also possible, to stimulate research and implementation of competency-based learning and approaches, for which probably often a reform of educational approaches is needed. The necessary financial resources to carry this out should be provided. Furthermore, in a climate where crime, violence and poor neighbourhood structure are predominant, governments should seek ways to provide appropriate environments for physical activity including, space, infrastructure and tools. Finally, governmental and non-governmental agencies in the region will do well to increase public health awareness of the importance of physical activity in mental health care. For example, physical activity should be integrated into the existing Information, Education and Communication (IEC especially on mental health and lifestyle diseases) public health awareness programs of the World Health Organization [53]. Targeted and regular messages should be developed in order to make this campaigns affordable. The benefits of engaging in physical activity should be properly outlined; any fears and wrongly held beliefs within various cultural contexts dispelled and dealt with, precautions to take as regards to starting at a low intensity and ways to maintain an active lifestyle should be included in the awareness programs across communities and policy makers.

## Conclusion

The current data shows that in SSA the importance of considering physical activity in mental health care is largely ignored in the current policies. Existing work force should be educated about the importance of an active lifestyle of their patients and should be trained in how to increase awareness in their patients. Policy makers should invest in qualified professionals to optimize mental and physical health improvements. It is known that inclusion of these professionals will improve outcomes and will ultimately without a doubt be cost-effective. Ignoring the potential of these professionals would be nothing less than a scandal, especially considering the increasing burden of mental health problems in this part of the world. Ministries of health and education in SSA play a critical role in stimulating research and improving training of existing work force and specialists. Furthermore, governments should seek ways to provide appropriate environments for physical activity and should increase public health awareness of the importance of physical activity for mental health. The evidence base of physical activity interventions in SSA is relatively limited but suggests that it can improve the health and wellbeing of this vulnerable population.

### What is known about this topic

- Mental and substance use disorders are the leading cause of years lived with disability in Sub-Saharan Africa;
- A mental health policy has been relatively low on the priority list in most countries of Sub-Saharan Africa;
- Physical activity interventions prevent the onset of mental illnesses while it can improve physical, mental and social health outcomes in people with established mental disorders.

### What this study adds

- Although physical activity is becoming acknowledged as a major component in the management of severe mental illness, the potential is yet to be embraced in Sub-Saharan Africa;
- Physical activity needs to be mainstreamed in existing health policies and strategies at all levels of intervention including primary health care;
- Professionals that can prescribe physical activity such as physical therapists should be given a role within the mental health care of people with mental illness.
